# Root Maturation of an Immature Dens Invaginatus Despite Unsuccessful Revitalization Procedure: A Case Report and Recommendations for Educational Purposes

**DOI:** 10.3390/dj11020047

**Published:** 2023-02-10

**Authors:** Julia Ludwig, Marcel Reymus, Alexander Winkler, Sebastian Soliman, Ralf Krug, Gabriel Krastl

**Affiliations:** 1 Center of Dental Traumatology, Department of Conservative Dentistry and Periodontology, University Hospital Würzburg (UKW), Pleicherwall 2, 97070 Würzburg, Germany; 2 Department of Conservative Dentistry and Periodontology, University Hospital, Ludwig-Maximilians-University Munich, Goethestrasse 70, 80336 Munich, Germany

**Keywords:** dens invaginatus, immature tooth, revitalization, apexification, 3D printing, dental education, replica

## Abstract

Background: The clinical management of teeth with complex dens invaginatus (DI) malformations and apical periodontitis may be challenging due to the lack of routine. The aim of this case report is to describe the endodontic treatment of an immature tooth with DI and to discuss strategies for preclinical training for teeth with such malformations. Case report: A 9-year-old male presented with an immature maxillary incisor with DI (Oehlers Type II) and apical periodontitis which was diagnosed by cone beam computed tomography (CBCT). Revitalization was initially attempted but then abandoned after failure to generate a stable blood clot. Nevertheless, considerable increase in both root length and thickness could be detected after medication with calcium hydroxide followed by root canal filling with MTA as an apical plug. Conclusions: The endodontic management of teeth with DI requires thorough treatment planning. In immature teeth, under certain conditions, root maturation may occur even with conventional apexification procedures. From an educational perspective, different strategies including CBCT and 3D-printed transparent tooth models for visualization of the complex internal morphology and redesigned 3D-printed replica with various degrees of difficulty for endodontic training, can be used to overcome the challenges associated with endodontic treatment of such teeth.

## 1. Introduction

Dens invaginatus (DI) describes a localized morphologic tooth anomaly, which is presumed to result from an infolding process of the dental papilla during tooth development [1]. The enamel is infolded into the dentine, creating a niche, which may contain organic material and may later be contaminated by bacteria from the oral cavity. The prevalence of this malformation was reported from 0.3 up to 10% in various populations [2]. The upper lateral incisors are most likely to be affected [3] while cases in canines, molars or even deciduous teeth are less common [4,5,6]. Its bilateral occurrence was also reported [2,7,8]. The most commonly used classification for DI was introduced by Oehlers in 1957. It divides cases into three categories, depending on the extent of the invagination [9]. In type I the invagination is minor and ends before the cement–enamel junction (CEJ). In type II the invagination extends into but is still confined within the root of the tooth. A dilation of the invagination and of the tooth and a communication of the invagination with the endodontic system of the pulp chamber can occur. In type III the invagination extends through the root of the tooth and can form a so-called second foramen either laterally or at the apex. Both the invagination and the tooth can be dilated. The invagination does normally not communicate with the pulp space and can be lined by a combination of enamel and cementum or solely by enamel [9].

If the invagination niche is colonized by bacteria, this can induce caries and further lead to infection of the root canal, pulp necrosis and apical periodontitis. Even before caries develops, interruptions in the invaginated enamel surface can allow penetration of microorganisms into the endodontic system [7].

The occurrence of apical periodontitis increases with increasing severity of the invagination. In a retrospective study on 4556 patients in a Turkish population 229 teeth with an invagination were found in 116 patients. Only one tooth of type I but 8% of type II and 88% of type III had apical periodontitis [10].

Since cases of DI can vary widely depending on the type and shape of the malformation, the treatment options are equally diverse and range from preventive sealing or “endodontic treatment” of the invagination cavity in a vital tooth without pulp infection, up to root canal treatment of both invagination and the true root canal system or even surgical therapy in teeth with pulp necrosis and apical pathosis [11,12]. Early diagnosis of the invagination is very important to prevent infection of the pulp by sealing the point of entry. If endodontic treatment of such a malformed tooth becomes necessary, the endodontic therapy can be challenging due to the unusual anatomy of the tooth, the endodontic system and the incorporated invagination itself. In immature teeth with apical periodontitis, revitalization procedures may induce further root formation and should be preferred [13].

While experienced and well-equipped endodontists can reliably manage the variety of treatment approaches, inexperienced clinicians may struggle when confronted with complex endodontic conditions [14]. Particularly, a lack of suitable training scenarios for endodontic procedures on teeth with anomalies can make the treatment of DI more difficult.

To overcome this problem, different approaches are conceivable in endodontic education. First, cone beam computed tomography (CBCT) is highly beneficial and recommended by the European Society of Endodontology for the appreciation of anatomically complex root canal systems associated with DI [15]. Second, transparent tooth models can be 3D-printed, in order to visualize the internal morphology of the tooth even better. Third, different replica of such teeth with various degrees of difficulty can be 3D-printed for endodontic training after redesign of the morphology of the invagination and of the root canal system to match the trainees’ experience level.

The aim of this case report was (i) to describe the treatment of an immature dens invaginatus with pulp necrosis and apical periodontitis and (ii) to discuss strategies for preclinical training for teeth with anomalies in need of endodontic treatment.

## 2. Case Report

This case report was prepared according to the PRICE 2020 Guidelines for reporting case reports in Endodontics [16] (Table A1).

### 2.1. Appointment 1

**Figure 1:**
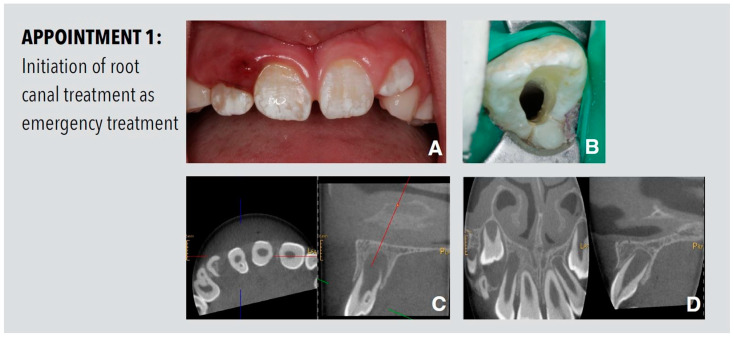
Procedures performed at the first appointment. (**A**) Preoperative clinical view of the maxillary front teeth showing swelling and redness of the gingiva in proximity to the right lateral incisor. (**B**) Initial access cavity. (**C**) Preoperative diagnostic CBCT shows the palatal invagination with partial enamel lining on tooth 12. (**D**) The sagittal view of the CBCT shows the periapical radiolucency associated with tooth 12 and arrested root development of tooth 12 in comparison to the unaffected tooth 22. The transversal projection of tooth 22 is shown on the right-hand side.

A 9-year-old male presented with pain in the anterior region of the upper jaw. The clinical inspection showed a localized swelling and redness of the buccal gingiva of the maxillary right lateral incisor. The tooth had a buccal probing depth of 9 mm and was tender on both apical palpation and percussion. There was no response to a pulp sensibility test with cold. A protuberance with a palatal localized fissure leading to the foramen caecum was detected. Cone beam computed tomography (CBCT) with a limited field-of-view revealed a periapical radiolucency associated with tooth 12, arrested root development and an isolated invagination, which extended up to the cement–enamel junction. A thin bridge of dental hard tissue between the invagination and the true endodontic system could be detected (Figure 1). Thus, the tooth was diagnosed a dens invaginatus of Oehlers Type II with pulp necrosis and apical periodontitis. After informed consent was obtained, the access cavity was prepared and disinfection of the endodontic system started (Figure 1). The root canal was irrigated with 3% sodium hypochlorite, dressed with calcium hydroxide paste (AH Temp, Dentsply Sirona, Konstanz, Germany) and temporized with Cavit W (3M, Seefeld, Germany).

### 2.2. Appointment 2

**Figure 2:**
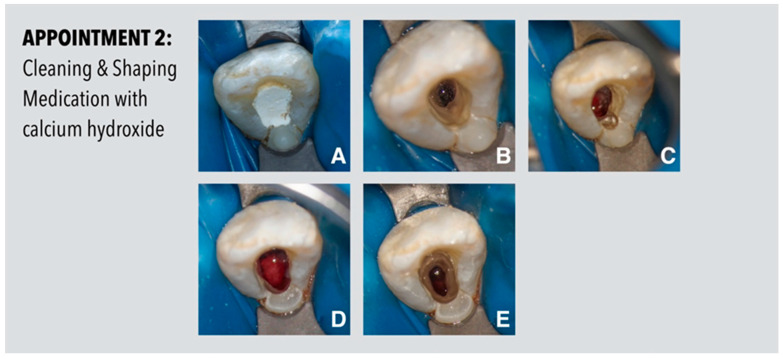
Procedures performed at the second appointment. (**A**) Temporized cavity suggesting a sufficiently extended access cavity. (**B**) After removal of the temporary material and inspection under the operating microscope a palatal overhang is visible: the invaginated tooth structure has not yet been incorporated into the access cavity. (**C**) Extension of the access cavity and removal of the invaginated tooth structures. (**D**) View of the fully extended access cavity with optimal access to the endodontic system. (**E**) Situation after disinfection and before placement of calcium hydroxide.

Four weeks later, the patient presented asymptomatic. The affected tooth showed normal responses to percussion and palpation and normal periodontal probing depths. The tooth was isolated by rubber dam and reopened (Figure 2). After careful inspection of the access cavity under the operating microscope and reexamination of the CBCT images it was noted that the extension towards the palatal side was insufficient and the invaginated tooth structure was not yet incorporated. Therefore, the access cavity was enlarged accordingly in order to remove the dental hard tissue on and around the invagination. Cleaning and shaping of the root canal was performed under irrigation with sodium hypochlorite (3%). A final irrigation protocol with EDTA (20%) and sodium hypochlorite in combination with a sonic irrigant activation tip, EDDY (VDW, Munich, Germany), was applied and a calcium hydroxide dressing was placed into the root canal. The access cavity was sealed with bonded composite resin (Filtek Supreme XTE Flow, 3M, Seefeld, Germany).

### 2.3. Appointment 3

**Figure 3:**
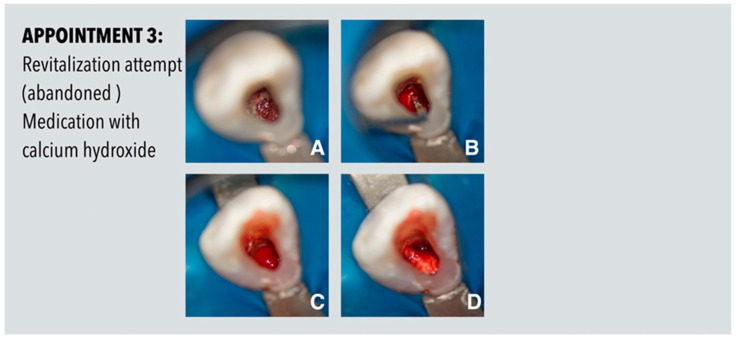
Procedures performed at the third appointment. (**A**) Situation after removal of calcium hydroxide. (**B**) Provocation of bleeding from the apex. (**C**) Persistent bleeding in the root canal. (**D**) No stable blood clot even after placement of collagen.

Due to scheduling reasons, further treatment was not carried out until eight weeks later. At this appointment, a revitalizing approach was performed according to the position statement of the European Society of Endodontology [13], but could not be completed due to persistent bleeding after provocation and the lack of a stable blood clot even after placement of collagen (Figure 3). As residual endodontic infection was assumed, the root canal was rinsed again with sodium hypochlorite (3%) in copious amounts and a calcium hydroxide dressing was placed again into the root canal. The access cavity was sealed again with composite resin (Filtek Supreme XTE Flow, 3M, Seefeld, Germany).

### 2.4. Appointment 4

**Figure 4:**
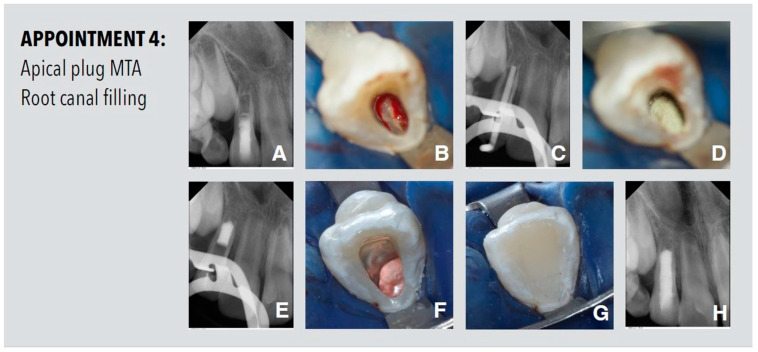
Procedures performed at the fourth appointment. (**A**) Periapical radiograph. (**B**) Hard tissue barrier was clearly visible under the operating microscope. (**C**) Radiographic working length determination with gutta-percha point. (**D**) Apical plug with MTA. (**E**) Radiologic view of the apical MTA plug up to the apical hard tissue barrier. (**F**) Obturated root canal using a warm vertical gutta-percha and sealer up to 3 mm below the cement–enamel junction (CEJ). (**G**) Sealed cavity with bonded composite resin. (**H**) Periapical radiograph of the root filled tooth.

After ten weeks, a hard tissue barrier could be detected at the apex of the root on the periapical radiograph (Figure 4). After irrigation and removal of calcium hydroxide remnants from the root canal, the incomplete apical hard tissue barrier was clearly visible under the operating microscope. The root canal was then dried using paper points and an apical MTA (ProRoot MTA; Dentsply Sirona, Konstanz, Germany) plug of 5 mm was placed by using an MTA gun (MAP System, Produits Dentaires SA, Vevey, Switzerland) and compacted with large paper points. The remaining canal space was obturated using a warm vertical gutta-percha and sealer up to 3 mm below the CEJ. The remainder of the root canal and access cavity was restored with a bonded composite material.

### 2.5. Recall Appointments

**Figure 5:**
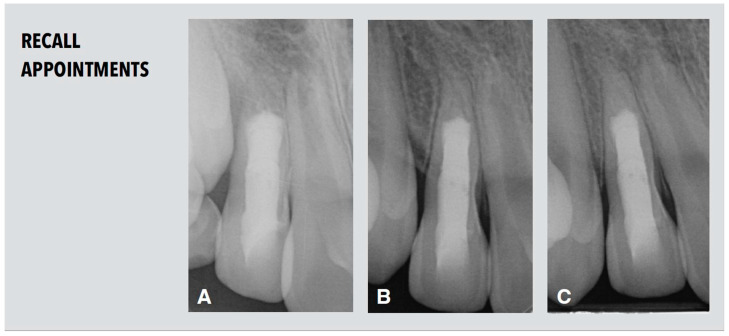
Radiological follow-up. (**A**) Periapical radiograph after nine months demonstrating increase in both root length and thickness in the apical part below the MTA level. (**B**,**C**) Periapical radiographs after two years (**B**) and after three years and nine month (**C**) demonstrating further root maturation.

The patient attended the first recall appointment nine months after root canal filling. The tooth remained asymptomatic. Healing of the apical lesion and a considerable increase in both root length and thickness in the apical part below the MTA level was visible in the periapical radiograph (Figure 5A). Further root maturation was detected two years after treatment (Figure 5B).

## 3. Fabrication of 3D Replica for Treatment Planning and Educational Purposes

**Figure 6:**
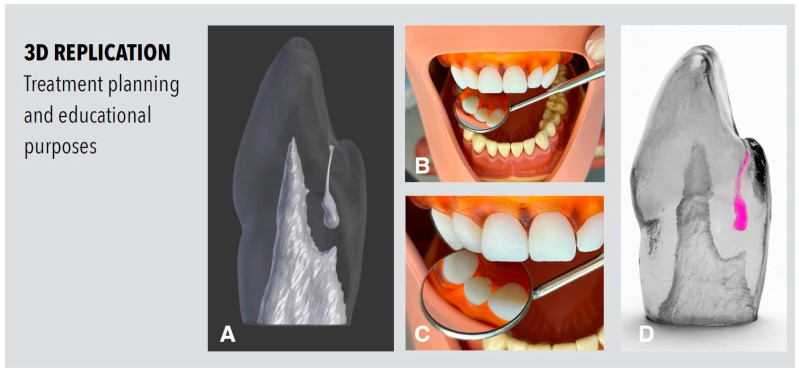
Three-dimensional replication for treatment planning and educational purposes. (**A**) Blender 3D view of the internal anatomy of an Oehlers Type II with a modified invagination. (**B**,**C**) Three-dimensional-printed replica of tooth 12 for clinical training scenarios in a tooth model and manikin head. (**D**) Transparent and enlarged 3D-printed tooth replica for visualization purposes of the internal root canal anatomy showing the exact location of the pink colored invagination niche.

The initial CBCT data set was segmented using the open-source software ITK-SNAP (Version 3.6.0, www.itksnap.org, accessed last on 6 February 2023), exported as an STL file and imported as a 3D image into the open-source 3D graphics software Blender, Version 2.78 (Blender Foundation, Amsterdam, The Netherlands). This offers a 3D view of the internal anatomy of the tooth and is the basis for possible digital modifications to simulate different situations and levels of difficulty using the Blender software and for 3D printing of replicas for endodontic training (Figure 6).

## 4. Discussion

There are numerous case reports on the treatment and the challenges associated with the endodontic management of teeth with dens invaginatus (DI) malformation and apical periodontitis [17,18,19,20,21]. Particularly when the invagination creates a very complex endodontic system, inadequate root canal disinfection may jeopardize healing. In the present case the initial endodontic access cavity appeared adequate in size and location at first glance, but the extension towards the palatal side was insufficient and the invaginated tooth structure was not yet incorporated. Adequate disinfection of the endodontic system was only possible after the access cavity had been enlarged accordingly. Further treatment challenges were associated with the immature root. In such cases the treatment should ideally promote the completion of root formation. To achieve this goal, revitalization procedures were recommended and the clinical protocol has been described in detail in the position statement of the European Society of Endodontology [13]. Provocation of bleeding by over-instrumenting is regarded as a key factor in order to generate a blood clot with stem cells from the apical papilla in the disinfected root canal as a basis for further root maturation [22].

In the present case, revitalization was initially attempted, but then abandoned after failure to generate a stable blood clot. Nevertheless, considerable increase in both root length and thickness could be detected after medication with calcium hydroxide followed by root canal filling with MTA as an apical plug. The radiographic appearance after two years resembled some typical cases published as successful revitalization in the literature [23]. In addition, the tooth meets all success criteria defined for revitalization procedures (absence of pain, swelling and sinus tract, resolution of apical radiolucency and root growth in length and thickness) [24] except for the lack of response to pulp vitality tests, which is a typical finding even after successful revitalization [23,25].

There is only scarce evidence suggesting that disinfection and calcium hydroxide medication without subsequent provocation of bleeding from the apex might also stimulate root formation in immature teeth [26,27]. A case series of twenty-one immature teeth with calcium hydroxide dressing followed-up for 14 to 75 months demonstrated complete root formation in fourteen and partial root formation in five teeth. Histologic material obtained from one tooth which was extracted due to horizontal root fracture in that study, revealed that considerable amounts of new tissue had been formed, both apically and within the old canal [28]. Although this case series was published more than five decades ago, these findings have hardly been noticed by the scientific community and it is still widely accepted that prolonged calcium hydroxide dressings in immature teeth, though leading to apical closure by hard tissue formation—do not allow for further root maturation [29,30]. This assumption seems to be supported by clinical studies, which could not demonstrate any significant increases in root length and particularly root width after this approach in contrast to revitalization procedures, which showed significant root maturation [31,32,33]. Therefore, a question arises as to why root maturation after calcium hydroxide medication but without implementation of a specific revitalization protocol as shown in this case report and in the above-mentioned case series from 1970 could not be confirmed in the more recent studies. One case report demonstrated that formation of the root apex occurred even without treatment in a case where a tooth with acute apical periodontitis, which remained open and contaminated by the oral environment for six months, still showed a development of root maturation in the radiograph [34]. Another case report demonstrated pulp revitalization after the periradicular disease was eliminated [35] and it is generally accepted that a viable Hertwig’s epithelial root sheath (HERS) is a prerequisite for further root formation. Thus, it might be speculated that the advanced and more aggressive disinfection protocols, such as irrigation with 5% sodium hypochlorite as is nowadays recommended and performed for conventional endodontic treatment of immature teeth [36,37], might have had an unfavorable effect on cellular structures in the HERS compared to the less aggressive approach with reduced irrigant concentration adopted in the 50-year-old case series [28]. Moreover, the position statement of the European Society of Endodontology [13] recommends irrigation with 1.5–3% sodium hypochlorite for revitalization procedures. However, in contrast to this, even though it is likely that higher concentrations of sodium hypochlorite can be detrimental to stem cells [38], another study found that the use of a 6% sodium hypochlorite concentration, in comparison to a lower solution of 1.5%, was associated with a greater increase in root development in a study on 51 cases of immature permanent teeth diagnosed with pulp necrosis, which were treated with regenerative endodontic procedures (REPs). They concluded that sufficient disinfection, the sodium hypochlorite concentration, the type of medication, the preoperative apical diagnosis and the etiology of pulp necrosis are key factors for the regenerative outcome in REPs [33]. In another case report, three cases of acute apical periodontitis on immature premolars were treated by a pulpotomy procedure because remaining vital pulp tissue could be observed clinically and led to the assumption that only a partial pulp necrosis was present, even though the patients showed distinct apical radiolucencies in the X-rays. Thus after removal of the infected portion of the pulp a recovery of the inflamed tissue at the apex of the roots could be detected, similar to findings of regenerative endodontic procedures [39]. More clinical research is needed to identify factors that might contribute to root maturation in conventional apexification procedures.

From an educational perspective, the present case is an ideal basis for the implementation of modern digital diagnosis, design and manufacturing techniques. This can be very beneficial as such cases are rare and not routinely treated in clinical practice, making it difficult to acquire clinical expertise.

Three-dimensional imaging techniques, such as CBCT, have already proven to be useful in complex cases of dens invaginatus [19,40,41]. They can help with accurate diagnosis and classification of the dens invaginatus, improve visualization of the morphology and internal anatomy of the tooth and can thus be crucial in determining the therapeutic strategy. Pradhan et al. concluded after treatment of a dens invaginatus Oehlers Type III, that the use of a CBCT image could prevent excessive loss of tooth structures [40].

In addition, a lack of suitable training scenarios for endodontic procedures on teeth with dental anomalies can make the treatment of dens invaginatus more challenging. The ideal options for endodontic training are extracted human teeth. However, to accumulate a selection of extracted teeth with specific dental anomalies seems highly unlikely due to their rarity. While commercial tooth replicas are available for the practice of common endodontic cases, no such replicas exist on the market for the simulation of rarer endodontic tooth anomalies like dens invaginatus. 

By using CBCT imaging data to create and print an exact replica of a tooth in need of endodontic treatment, a case specific training scenario can be created so that treatment can be approached on the replica first, and then the actual treatment can be performed on the patient’s tooth later on. In a complex case of Oehlers Type III invagination on a vital tooth with bacterial infection confined to the invaginated part of the tooth, Kfir et al. produced three-dimensional plastic replicas of the tooth based on the information obtained through a CBCT scan [19]. By using a transparent 3D-printed replica the anatomy of the tooth was studied, the therapeutic approach chosen and the direction and depth of the access for the root canal treatment of solely the invagination were planned. By using another opaque replica the practitioner was able to extensively practice the root filling procedure until sufficient skills were acquired for the clinical procedure. The placement of the MTA plug into the invagination cavity was practiced on the tooth replica and monitored by X-ray until the desired result could be reproducibly generated [19]. This shows that the use of printed tooth replicas can actually help the practitioner to become familiar with the complex internal anatomy of the tooth and facilitate clinical performance with more safety during endodontic procedure. As a further technical development, endodontic treatment of teeth with dens invaginatus [42] and dens evaginatus malformation [43] could be meticulously planned and performed with the guided endodontics approach [44,45].

However, such case-based workflows require not only readily available equipment but can also be time consuming and might not be feasible in clinical practice. In the present case, the patient presented with pain and needed immediate treatment. 

Therefore, in addition to individualized case-based training and specific technical aids, it would be desirable to have autonomous training scenarios, which enable the practice of endodontic procedures on teeth with different variations of the malformation. By using 3D design software, it is possible to either design from scratch or to modify the data set of an existing tooth or root canal and implement a range of variations for different training scenarios. If three-dimensional imaging using CBCT is available, the data set can be segmented, exported as an STL file and modified in different ways. Many software solutions are available for this purpose, for example Blender (blender.org.). Several dental schools now use 3D printers to print teeth for training purposes and create individualized scenarios. By designing the external and internal anatomy, the natural variations of human teeth can be replicated with varying degrees of difficulty. This would allow for a skill-based training, practicing first on an easier tooth replica and later on a more challenging one.

Despite the documented benefits of 3D-printed teeth for practical training in endodontics, the material properties of the replicas are still the biggest drawback since no commercial material seems able to simulate dental hard tissues in every desired aspect, particularly concerning hardness and radiopacity [46,47,48]. This is particularly disadvantageous for teeth with such malformations, because in a clinical situation the enamel lining of deep invaginations is a clearly perceptible obstacle while negotiating the endodontic system, which cannot be easily replicated yet with usual 3D-printing technology. Further development is needed in matching the material properties of the 3D-printed replicas with those of natural teeth.

Still, the use of printed replicas and modified versions of different invaginations in degrees of difficulty seems to be a feasible training option in preparation of clinical endodontic procedures on teeth with different malformations and could be applied to student courses and during more advanced endodontic training. Furthermore, the technique of modification could be applied to different endodontic scenarios.

## 5. Conclusions

The clinical management of teeth with dens invaginatus malformation and apical periodontitis may benefit from different strategies including CBCT and 3D-printed transparent tooth models for visualization of the complex internal morphology. Redesigned 3D-printed replicas with various degrees of difficulty for endodontic training can be used to overcome the challenges associated with endodontic treatment of such teeth. 

In immature teeth, under certain conditions, root maturation may occur even with conventional apexification procedures.

## Data Availability

Not applicable.

## Appendix A

**Table A1:** PRICE 2020 checklist of the present case report.

Section/Topic	Item Number	Checklist Item	Reported on Page Number
**Title**	1a	The words “case report(s)” must be included in the title	1
	1b	The area of interest (e.g., anatomy, disease, treatment) must be included briefly in the title	1
**Keywords**	2a	At least two relevant keywords, preferably MeSH terms, related to the content of the case report must be included	1
**Abstract**	3a	The Introduction must contain information on how the report is novel and contributes to the literature, clinical practice and/or fills a gap(s) in knowledge	1
	3b	The Body must describe the main clinical findings, including symptoms and signs, if present	1
	3c	The Body must describe the main radiographic/histological/ laboratory/diagnostic findings	1
	3d	The Body must describe the main outcomes of treatment, if active treatment has been provided	1
	3e	The Conclusion(s) must contain the main “take-away” lesson(s), sometimes referred to as key learning point(s)	1
**Introduction**	4a	A background summary of the case(s) with relevant information must be provided	1–2
**Informed consent**	5a	A clear statement that informed, valid consent was obtained from the patient(s) must be provided	2
**Case report information**	6a	The age of the patient(s) must be provided	2
	6b	The gender of the patient(s) must be provided	2
	6c	The ethnicity of the patient(s) must be provided, if relevant	N/A
	6d	The main concern, chief complaint or symptoms of the patient(s), if any, must be provided	2
	6e	The medical history of the patient(s) must be provided, if relevant	N/A
	6f	The dental history of the patient(s) must be provided, if relevant	N/A
	6g	The family history of the patient if associated with the primary complaint must be provided, if relevant	N/A
	6h	The psychosocial history of the patient if associated with the primary complaint must be provided, if relevant	N/A
	6i	Genetic information, including details of relevant comorbidities and past interventions and their outcomes must be provided when possible, if relevant	N/A
	6j	Extra-oral findings must be provided, if relevant	N/A
	6k	General intra-oral findings must be provided when relevant, e.g., carious lesions, restorations, periodontal condition, soft tissues, etc.	2–3
	6l	Important/relevant dates and times (in the text, or a table or figure) must be provided in chronological order	2–5
	6m	The diagnostic methods and the results for the specific tooth/teeth (e.g., pulp sensibility test, tenderness, mobility, periodontal probing depths, laboratory investigations, imaging techniques, or other special tests) must be provided	2–5
	6n	The diagnostic challenges, if any, must be provided	N/A
	6o	The diagnostic reasoning including other possible diagnoses that were considered must be provided	N/A
	6p	The active treatment (s) or intervention(s) performed, if any, must be provided	2–5
	6q	Any modifications to the proposed treatment(s) or intervention(s), if necessary, must be provided	4–5
	6r	The assessment method(s) used to determine the clinician-assessed and patient-assessed treatment outcomes and their results must be provided	5
	6s	Adverse and unanticipated events or consequences, if any, must be provided	N/A
**Discussion**	7a	The specific treatment(s) and intervention(s) (if any) must be discussed with reference to the relevant literature	6–7
	7b	The strengths of the case report and its importance must be discussed with reference to the relevant literature	6–8
	7c	The limitations of the case report must be discussed	6–9
	7d	The rationale for the conclusion(s) must be discussed	6–9
**Patient perspective**	8a	Feedback from the patient on the treatment and the care they received should be provided, if relevant	N/A
**Conclusion**	9a	Explicit conclusion(s), i.e., the main “take-away” lessons must be provided	9
	9b	Implications for clinical practice or future research must be provided	9
**Funding details**	10a	Sources of funding and other support (such as supply of instruments, equipment) as well as the role of funders must be acknowledged and described	N/A
**Conflict of interest**	11a	An explicit statement on conflicts of interest must be provided	9
**Quality of images**	12a	Details of the equipment, software and settings used to acquire the image(s) must be described in the text or legend	N/A
	12b	The reason why the image(s) was acquired and the rationale for its inclusion in the manuscript must be provided in the text	N/A
	12c	The circumstances (conditions) under which the image(s) were viewed and evaluated by the authors must be provided in the text	N/A
	12d	The resolution and any magnification of the image(s) or any modifications/enhancements (e.g., adjustments for brightness, color balance, or magnification, image smoothing, staining, etc.) that were carried out must be described in the text or legend	N/A
	12e	Patient(s) identifiers (names, patient numbers) must be removed to ensure they are anonymized	N/A
	12f	An interpretation of the findings (meaning and implications) from the image (s) must be provided in the text	3–6
	12g	The legend associated with each image must describe clearly what the subject is and what specific feature(s) it illustrates. Legends associated with images of patients must describe the age, gender and ethnicity of the person, if relevant	3–6
	12h	Markers/labels must be used to identify the key information in the image(s) and be defined in the legend or as a footnote	N/A
	12i	The legend of each image must include an explanation whether it is pre-treatment, intra-treatment or post-treatment and, if relevant, how images over time were standardized	3–6

N/A: not applicable.
